# Student participation in accelerated experience-based co-design for healthcare decarbonisation education: a focused ethnographic study

**DOI:** 10.1186/s12909-026-10015-1

**Published:** 2026-07-25

**Authors:** Nuala McLaughlin-Borlace, Stephanie Craig, Tara Anderson, Nuala Flood, Laura Steele, Tracy Levett-Jones, Fadwa Alhalaiqa, Dalal Hammoudi Halat, Norfadzilah Ahmad, Jesus Sánchez-Martín, Charlotte McArdle, Naomi Tutticci, Sara Lynch, Rose Gallagher, Gary Mitchell

**Affiliations:** 1 School of Nursing and Midwifery, Queen’s University Belfast, Belfast, UK; 2 School of Natural and Built Environment, Queen’s University Belfast, Belfast, UK; 3 Queen’s Business School, Queen’s University Belfast, Belfast, UK; 4 School of Nursing and Midwifery, University of Technology Sydney, Ultimo, Australia; 5 Pre-Clinical Affairs, College of Nursing, Qatar University, Doha, Qatar; 6 QU Health Office of Assessment and Accreditation, QU Health, Qatar University, Doha, Qatar; 7 Faculty of Nursing, International Islamic University Malaysia, Kuantan Campus, Kuantan, Pahang Malaysia; 8 Facultad de Educación y Psicología, Universidad de Extremadura, Badajoz, Spain; 9 Faculty of Health Science, Ulster University, Londonderry, UK; 10 School of Nursing and Midwifery, Griffith University, Brisbane, Australia; 11 Estates Directorate, Queen’s University Belfast, Belfast, UK; 12 Royal College of Nursing, London, Greater London, UK

**Keywords:** Co-design, Healthcare decarbonisation, Health professions education, Digital education, Sustainability

## Abstract

**Background:**

Pre-registration health professional students receive limited education on sustainable clinical practice within healthcare. Education on healthcare decarbonisation, a key strategy for reducing the environmental impact of healthcare delivery, also remains underdeveloped within many curricula. Co-designed e-resources offer a practical approach to help address this gap; however, their effectiveness depends on being relevant, engaging, and grounded in students’ experiences. This study aimed to co-design an e-resource on healthcare decarbonisation using an Accelerated Experience-Based Co-Design (AEBCD) approach with an embedded focused ethnography.

**Methods:**

An adapted four stage AEBCD approach was used. Four co-design workshops were conducted over four months with interdisciplinary health profession students (nursing and pharmacy). An embedded focused ethnography captured social, cultural, and interactional dynamics through observational researcher field notes. Artefacts and post-workshop focus groups informed iterative development and explored participants experiences of co-design. Field notes were analysed using Rapid Qualitative Inquiry (RQI) and focus groups were analysed thematically.

**Results:**

Fifteen pre-registration nursing and pharmacy students participated in the workshops. The co-design process resulted in a four-module gamified e-resource on healthcare decarbonisation. Three overarching themes were identified during post-workshop focus groups were: (1) increasing agency and confidence in action, (2) fostering interdisciplinary collaboration and shared purpose, and (3) the value of structured facilitation and tangible outputs. Students reported enhanced engagement, ownership, and perceived readiness to apply sustainability principles in future professional practice.

**Conclusion:**

The AEBCD approach provided a robust and participatory framework for co-designing a learner-centred e-resource on healthcare decarbonisation. The integration of focused ethnography strengthened understanding of how co-design processes shaped engagement and decision-making, ensuring that stakeholder experiences directly informed both content and delivery. This combined methodological approach offers a transferable process for integrating AEBCD with focused ethnography, strengthening the visibility, reflexivity, and reproducibility of co-design in health professions education.

**Supplementary Information:**

The online version contains supplementary material available at https://doi.org/10.1186/s12909-026-10015-1.

## Introduction

Healthcare systems contribute substantially to global greenhouse gas (GHG) emissions, with hospitals, primary care services, pharmaceuticals, medical technologies, and complex global supply chains accounting for approximately 4–5% of total global emissions [1–3]. These emissions arise from energy-intensive infrastructure, resource consumption, waste generation, carbon-intensive procurement and clinical practices. As the climate crisis accelerates, the health sector faces a dual challenge: responding to the growing health impacts of climate change while simultaneously reducing its own contribution to environmental harm [4, 5]. In this context, healthcare decarbonisation has emerged as both an environmental imperative and a professional responsibility [6, 7]. Healthcare decarbonisation refers to efforts to reduce GHG emissions across healthcare systems while maintaining high quality care. This includes improving energy efficiency, transitioning to renewable energy, reducing waste, embedding low-carbon clinical pathways, influencing staff behaviours and organisational cultures, and decarbonising supply chains through sustainable procurement [8–10]. Importantly, decarbonisation strategies can generate co-benefits for health systems and populations, including improved air quality, cost savings, and enhanced resilience of healthcare infrastructure [11, 12].

There is increasing international consensus that achieving net-zero healthcare systems requires action at all levels, notably strong leadership, policy alignment, technological innovation, and workforce engagement [3, 6, 13]. Clinicians and future health professionals are increasingly recognised as critical agents of change, given their influence on clinical decision-making, resource use, and organisational culture [6]. However, meaningful engagement in decarbonisation requires more than awareness; it demands knowledge, practical skills, confidence, and a sense of professional responsibility for sustainable practice, reflecting the need for systems-based approaches that integrate behavioural and organisational change [14].

Despite this, education on healthcare decarbonisation remains limited, fragmented, and inconsistently embedded within pre-registration health professions curricula [15]. Where sustainability content is included, it is often delivered through didactic, knowledge-focused approaches [16–18]. Students frequently report feeling underprepared to address sustainability challenges in clinical settings [19]. Recent evidence further highlights the need for educational approaches that move beyond awareness-raising to develop practical and action-oriented learning in sustainability and decarbonisation [14]. Digital educational resources offer a scalable and flexible means of supporting this shift, particularly within undergraduate programmes where timetabling constraints, variable prior knowledge, and large cohorts require accessible learning approaches. When designed effectively, digital resources can support interactive, self-directed, and applied learning with clinical practice, while reducing the environmental impact of resource-intensive educational delivery methods [20, 21]. Existing educational resources on healthcare decarbonisation are rarely co-designed with learners. Although the benefits of co-design in health professions education are well established, many resources lack structured co-design methodologies and explicit theoretical grounding [22]. Together, this highlights a dual gap: the lack of learner-centred, co-designed educational resources focused on healthcare decarbonisation, and a limited understanding of how participatory design methods can be effectively operationalised within health professional education.

Co-design methodologies offer a promising solution to this gap. Co-design actively involves end users and stakeholders in developing interventions that reflect lived experience, learning needs, and real-world challenges [23, 24]. In health professions education, it has empowered students, patients, and researchers to collaboratively shape curricula, enhancing relevance, ownership, and alignment with professional practice [25–27]. However, co-design processes are often poorly described, limiting transferability and methodological learning, and risk tokenism when collaboration is insufficiently articulated or reflexively examined [28–32].

Experience-Based Co-Design (EBCD) uses narrative accounts to identify key “touchpoints” for improvement, while Accelerated Experience Based Co-Design (AEBCD) streamlines this process using existing resources, retaining core principles such as storytelling, iterative feedback, and collective prioritisation [33]. AEBCD has demonstrated feasibility across healthcare contexts and is increasingly used where resources or timelines preclude full EBCD cycles. Within health professions education, AEBCD offers a structured yet flexible framework for developing digital learning interventions that are contextually relevant and grounded in learners’ lived experiences [34, 35]. To address persistent critiques that co-design studies insufficiently attend to process, context, and interactional dynamics, this study integrates a focused ethnography alongside the AEBCD approach. Focused ethnography is well suited to short-term, intensive research engagements that aim to understand specific practices, interactions, and cultural norms within bounded settings [36–38]. By embedding observation, reflexive fieldwork, and rapid analytic cycles within the co-design process, focused ethnography enables deeper insight into how group dynamics and power relations shape design decisions and participant engagement [39]. Combining AEBCD with focused ethnography therefore responds directly to calls for more transparent, theoretically informed, and reflexive applications of co-design in health and education research [28, 29].

This study makes a primary methodological contribution by demonstrating how AEBCD can be integrated with focused ethnography and rapid qualitative approaches to enhance the transparency and rigour of co-design processes, while illustrating the development of an e-resource on healthcare decarbonisation.

### Aim and objectives

The aim of this study was to co-design an e-resource on healthcare decarbonisation using an AEBCD approach with pre-registration health professional students, and to examine the co-design process through an embedded focused ethnography.

The objectives of this paper were to:

1. Describe the application of an AEBCD approach in the development of an e-resource on healthcare decarbonisation.
2. Use focused ethnographic observations and post-workshop focus group data to explore student engagement, interaction, and participation across the co-design process.

This study asked the research question, “How can an AEBCD approach, informed by focused ethnography, be applied to co-design an e- resource on healthcare decarbonisation for pre-registration health professional students, and what findings emerge from students’ experiences of participating in the co-design process?”. The Guidance for Reporting Involvement of Patients and the Public (GRIPP2) checklist has guided study reporting [40] (*Supplementary File 1*).

## Method

### Research design

This study employed an AEBCD methodology, underpinned by principles of participatory action research, user-centred design, and narrative-driven engagement [33, 41]. AEBCD was selected to ensure that the resulting intervention was contextually relevant, pedagogically meaningful, and grounded in student experience, while remaining feasible within a doctoral research programme. Co-design actively involves stakeholders as partners in the design process, ensuring that interventions reflect users’ needs, priorities, and values [23, 42]. Within the taxonomy of co-design approaches, EBCD is distinguished by its use of experiential narratives to identify emotionally salient “touchpoints” that shape priorities for improvement [43]. While EBCD has been widely applied in healthcare service redesign [44], it’s increasingly recognised as valuable in educational design [45, 46]. AEBCD adapts the original EBCD framework by replacing prolonged, locally conducted ethnographic interviews with pre-existing, rigorously collected narrative data, enabling a more time-efficient process [33]. This approach has demonstrated feasibility and effectiveness across a range of applied health contexts, including service improvement and intervention development [35, 39]. To strengthen methodological rigour and capture the social and cultural dynamics of the co-design process, focused ethnography was embedded throughout the AEBCD workshops. Focused ethnography offers a pragmatic alternative to traditional ethnography, characterised by short-term, intensive field engagement and concentrated examination of specific practices within defined settings [47, 48]. This approach is particularly suited to time-limited, intervention-oriented research, where understanding interactional processes, power dynamics, and contextual factors is essential but prolonged immersion is neither feasible nor necessary [49, 50]. These adaptations align with frequently reported modifications to EBCD and AEBCD, including the use of audio recorded focus groups rather than individual interviews and removal of prolonged observation phases [35]. Adaptations were considered and documented to balance fidelity to core EBCD principles with pragmatic flexibility [51]. An overview is presented in Table 1.

**Table 1:** Overview of methodological components and their roles within the study

Component	Role in Study	Purpose	Stage of Application
Accelerated Experience-Based Co-Design (AEBCD)	Design framework	Structured the co-design process, including workshops, trigger materials, and participatory activities	Overall study design (Stages 1–4)
Focused ethnography	Process lens	Examined interactional dynamics, participation, and co-design processes in real time	Embedded across all workshops (Stages 3–4)
Rapid Qualitative Inquiry (RQI)	Iterative analytic approach	Enabled rapid analysis of field notes to inform ongoing workshop refinement and facilitation	Ongoing cycles between workshops (Stages 3–4)
Reflexive thematic analysis	Interpretive analytic method	Generated themes from focus group data to understand student experiences of co-design	Post-workshop analysis (Stages 3–4)
GRIPP2 checklist	Reporting framework	Supported transparent reporting of stakeholder involvement and co-design processes	Study reporting

### Ethical considerations

The study received ethical approval from the Faculty of Medicine, Health and Life Sciences Research Ethics Committee at Queen’s University Belfast on the 26 June 2025 (Reference: MHLS 25_96) and was conducted in accordance with the Declaration of Helsinki [52]. All participants received written study information and provided written informed consent, including consent for the use of photographs for research and dissemination purposes. Participation was voluntary and participants could withdraw at any time; however, withdrawal of individual focus group data was not possible once data had been anonymised and incorporated into collective analysis. Data were securely stored and managed in accordance with institutional policy.

### Recruitment

Three workshops and three focus groups were conducted in person at a UK university. The final workshop and focus group were held online via MS Teams. Workshops and focus groups were conducted over a four-month period (October 2025 - January 2026). The study was open to all pre-registration health professional students across nursing, midwifery, medicine, pharmacy, and dentistry. Participants were recruited using non-random convenience sampling, facilitated via Directors of Education within health professions’ schools at Queen’s University Belfast who acted as gatekeepers to the study; therefore, the total number of students who received invitations could not be determined. Consistent with co-design methodology, recruitment aimed to establish a manageable participatory group rather than a representative sample. While the study was open to multiple disciplines, only students from nursing and pharmacy expressed interest and participated in the workshops (Table 4). *An International Observer Panel (IOP) of climate and sustainability experts across academic*,* research*,* practice*,* policy*,* and non-healthcare disciplines provided transdisciplinary input throughout the study. Members were purposively selected based on their recognised expertise and experience across these areas to ensure broad academic*,* professional*,* and policy perspectives informed the study. The IOP offered strategic guidance*,* validated trigger materials*,* informed study design and workshop planning*,* and contributed to interpretation and manuscript development*,* but did not participate directly in the co-design workshops.*

### Integrated AEBCD and focused ethnography process

**Fig. 1:**
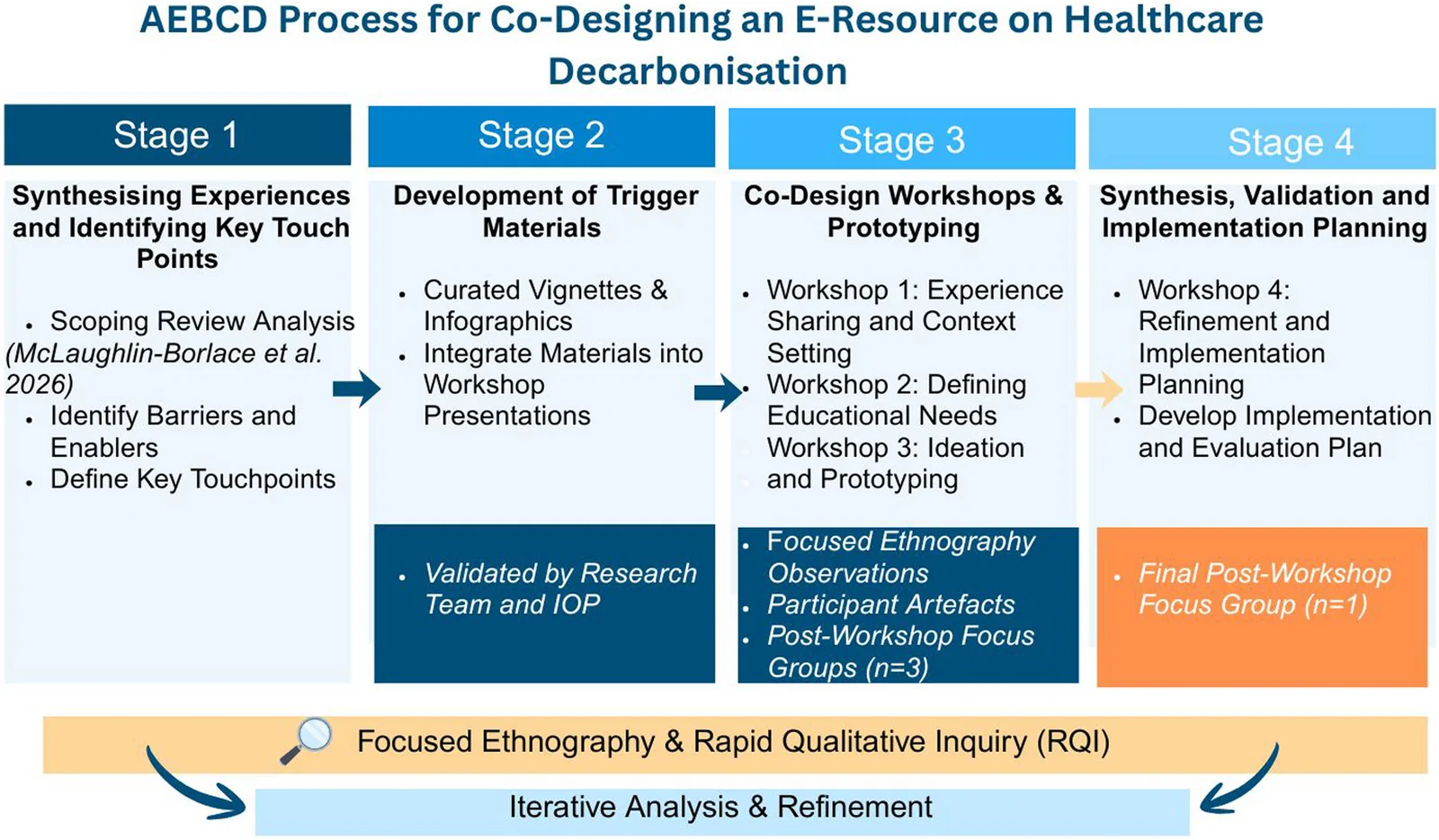
AEBCD Stages

In line with established AEBCD guidance [33] the study followed an adapted four-stage process to develop an e-resource on Healthcare Decarbonisation (Fig. 1).

Stage 1 involved synthesising experiences and identifying key touchpoints using findings from a prior scoping review [15] in place of primary ethnographic interviews. This review identified key barriers including knowledge gaps, limited confidence and agency, eco-anxiety, and difficulties applying sustainability principles in practice. Enablers included interactive, context-specific learning, reflection, collaboration, role modelling, and institutional support (Table 2). These findings informed Stage 2, guiding the development of trigger materials (Supplementary 2), workshop activities, discussion prompts, and prioritisation exercises to ensure the co-design process reflected learners’ experiences and educational needs.

**Table 2:** Summary of Stage 1 Touchpoints (McLaughlin-Borlace et al. 2026

Domain	Key Findings	Enablers / Recommendations
Knowledge & Understanding	Gaps in foundational knowledge of sustainability and healthcare decarbonisation	Interactive, context-specific teaching; blended and technology-enhanced learning
Confidence & Agency	Limited confidence in applying sustainability principles to clinical practice	Opportunities for practice, feedback, and reflective/collaborative learning; longitudinal embedding of content
Emotional Dimensions	Eco-anxiety and other emotional responses	Address emotional aspects explicitly in curricula; model sustainable behaviours
Systemic & Cultural Barriers	Structural constraints, hierarchical and institutional factors limiting action	Alignment with institutional priorities, visible role modelling, and practical demonstration in clinical contexts
Learning Approaches	Single-session or didactic approaches insufficient	Longitudinal, scaffolded learning; hands-on practice; interdisciplinary and collaborative exercises
Evaluation & Outcomes	Few studies measured decarbonisation-specific outcomes; reliance on self-reported data	Use controlled, longitudinal, and mixed-methods designs; link learning to measurable clinical sustainability practices

2.) Stage 2 involved the development of trigger materials (*Supplementary 2*) including vignettes derived from Stage 1 findings (Table 2), short films, and infographics were integrated into a presentation to use during the AEBCD workshops to build capacity, prompt reflection, and stimulate discussion and idea generation. Trigger materials were included if they were credible, relevant to healthcare decarbonisation, appropriate for pre-registration health professional students, and capable of stimulating reflective discussion, and excluded if they lacked relevance, credibility, accessibility, or pedagogical suitability. Trigger materials were reviewed and validated by the research team (N.M, G.M, S.C, N.F, L.S, T.A) and the IOP (T.L.J, F.A, D.H, N.B.A, J.S.M, C.M, N.T, S.L, R.G) via an independent review and consensus process in line with inclusion exclusion criteria (Table 3) to ensure scientific accuracy and pedagogical relevance.

**Table 3:** Inclusion/Exclusion Criteria for Trigger Materials

Included	Excluded
Relevant to climate change and healthcare decarbonisation	Unrelated to sustainability or healthcare
Credible academic, professional, or public-sector sources	Non-credible or commercial sources
Appropriate for pre-registration health students	Overly technical or unsuitable for undergraduate level
Designed to prompt reflection and discussion	Didactic content with limited interactive potential
Open-access multimodal formats (e.g., videos, infographics)	Inaccessible or non-open-access materials

**Fig. 2:**
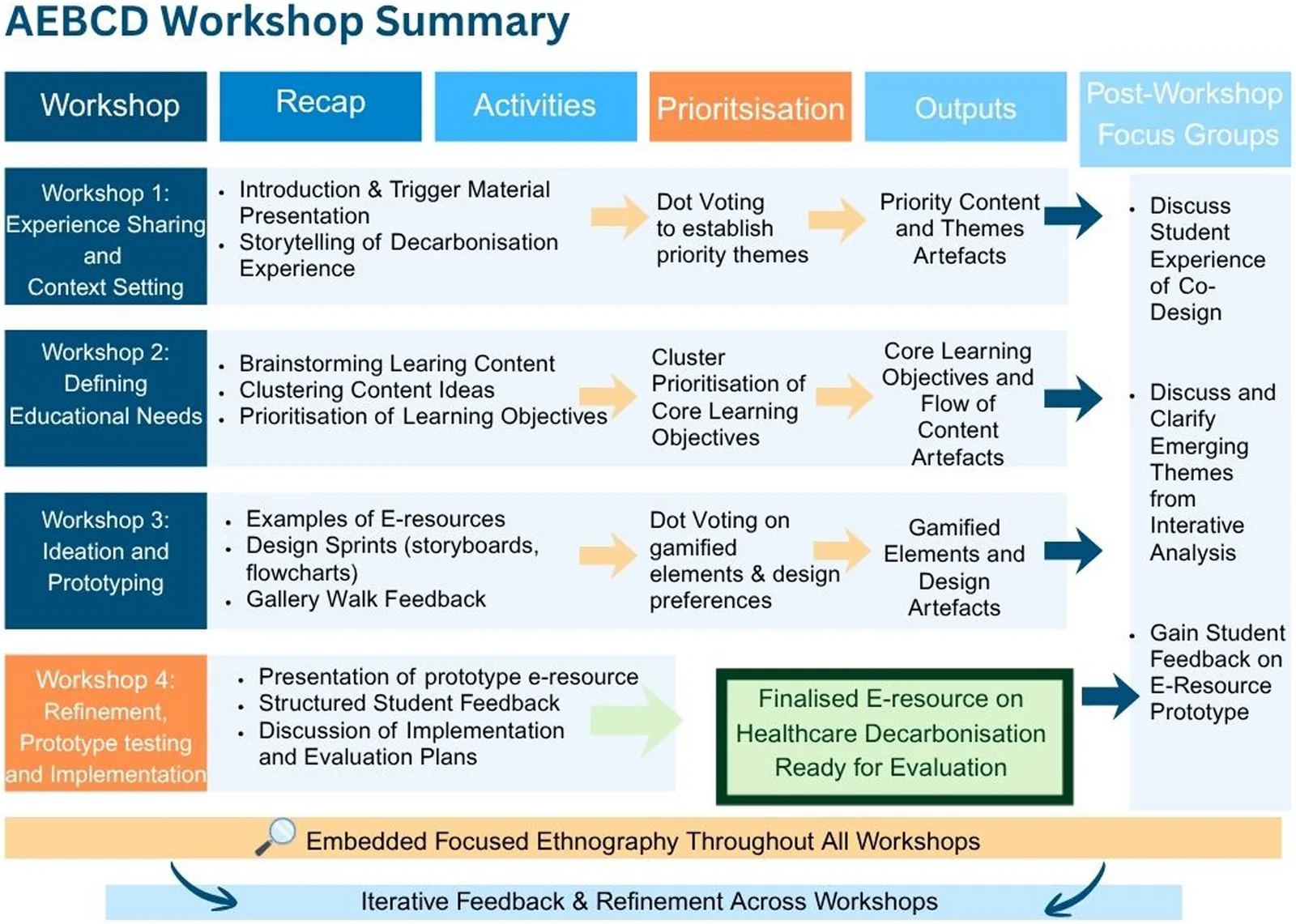
AEBCD Workshop Summary

3.) Stage three of the study involved a structured series of four workshops designed to engage pre-registration health professional students (*n* = 15) in the co-design of a e-resource on healthcare decarbonisation (Fig. 2). Each workshop was designed to build iteratively on the outputs of the previous session, ensuring that student perspectives directly informed both the content and structure of the e-resource. The workshops integrated trigger materials, group activities, and prioritisation exercises, and were followed by post-workshop focus groups (*n* = 4) to capture participants’ experiences and reflections. Workshops lasted approximately 3 h and were facilitated in the evening to maximise accessibility for students across differing academic timetables. A focused ethnographic approach was embedded across all workshops to examine student interactional processes and participation dynamics. Ethnographic observations recorded by researcher field notes were analysed via RQI and informed iterative refinement of workshop facilitation and co-design activities, ensuring reflexive and responsive engagement with participants. Focus groups were audio-recorded with participants’ consent and transcribed verbatim for analysis.

#### Workshop 1: experience sharing and context setting

**Fig. 3:**
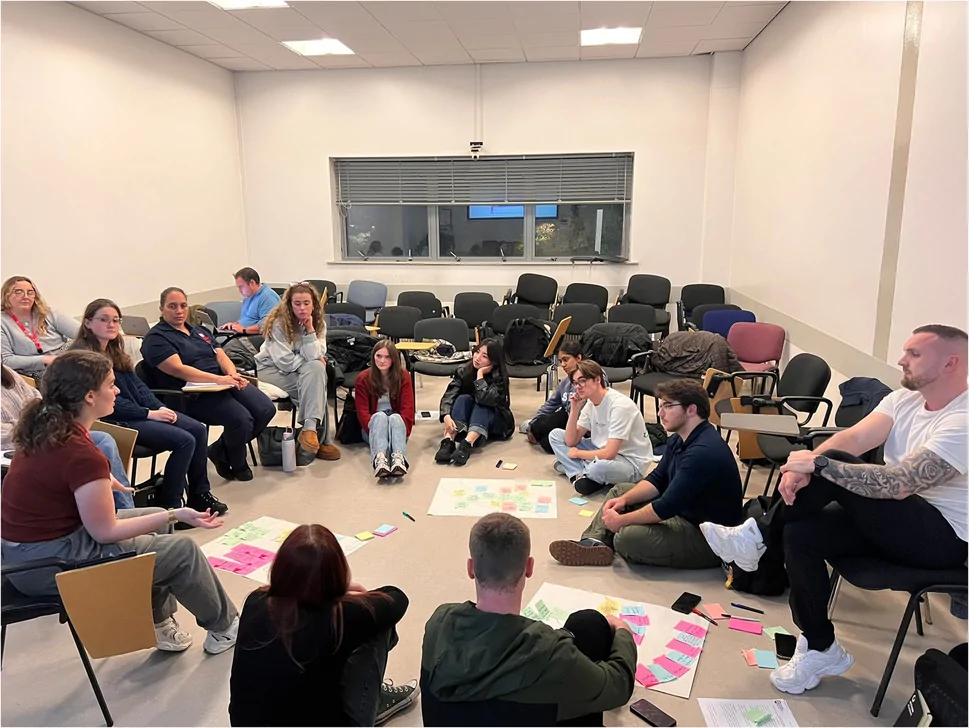
Co-design workshop (participants thematic clustering activity) – McLaughlin-Borlace (2025)

Workshop 1 focused on capacity building and developing a shared understanding of healthcare decarbonisation. Following engagement with trigger materials, students explored their existing knowledge, beliefs, and experiences through thought-cloud, small group storytelling (3–4 participants), and experience journey mapping activities. Using colour-coded sticky notes, participants identified barriers (red), emotions (blue), ideas (yellow), and potential solutions (green), before prioritising key challenges through a dot-voting exercise (Fig. 3). Focused ethnographic observation examined early participation and interactional dynamics, while a post-workshop focus group captured participants’ reflections on the co-design process. Findings informed the priorities and activities for Workshop 2.

#### Workshop 2: defining educational needs

Building on the priorities identified in Workshop 1, the second workshop began with a recap of the group priorities carried forward through dot-voting. Mixed-discipline groups (3–4 participants) engaged in structured brainstorming activities to explore potential e-resource content, focusing on the questions: “Where should the e-resource start and end?” and “What key knowledge and skills should be included?”. Groups shared their ideas, which were then thematically clustered to identify core learning objectives, supplementary content, and broader modules, while also determining the overall flow of the e-resource. Iterative discussion ensured alignment across disciplines and supported content prioritisation. Focused ethnographic field notes explored evolving group dynamics, facilitator-participant interactions, and engagement with co-design activities, while documenting the influence of iterative refinements (e.g., name badges, structured recaps, and optional preparatory materials) on participation. The workshop concluded with a focus group exploring participants’ experiences of the co-design process and emerging themes. The outcomes of Workshop 2 directly informed the design concepts developed in Workshop 3.

#### Workshop 3: ideation and prototyping

**Fig. 4:**
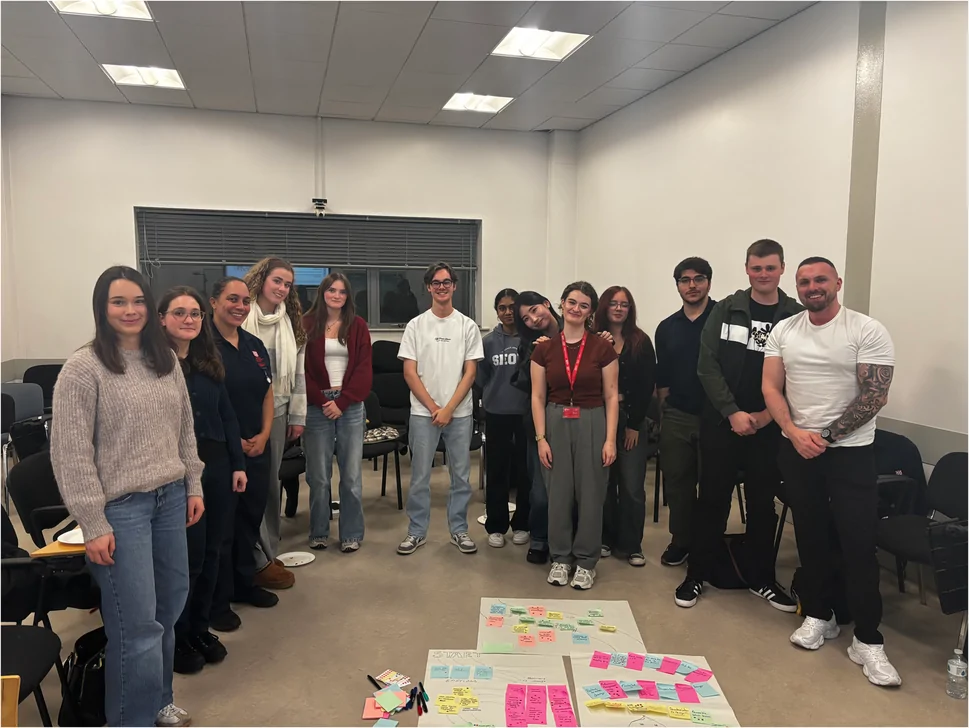
Co-design workshop (group photo) - McLaughlin-Borlace (2025)

The third workshop began with a recap of priorities from Workshop 2 and incorporated participant feedback from previous focus groups, including the continued use of group activities, preparatory materials, and name badges. Students explored examples of existing gamified and digital e-resources to generate ideas about structure, interactivity, and engagement. Working in small groups (3–4 participants), they undertook design sprints to develop prototypes (e.g., storyboards and flowcharts) representing proposed navigation, gamified elements, and content structure. Prototypes were shared through a gallery walk, enabling peer feedback, annotation, and refinement (Fig. 4). Facilitators synthesised emerging ideas into a coherent e-resource design, while a final discussion and dot-voting exercise established consensus on key design features and gamified elements. Focused ethnographic observation examined collaborative design processes, negotiation of ideas, and interactional dynamics during prototyping and feedback activities. The workshop concluded with a focus group exploring participants’ reflections on the evolving co-design process.

##### Workshop 4: refinement and implementation planning

The fourth workshop focused on the presentation of an initial draft e-resource via an online workshop hosted on MS Teams. Facilitators recapped the journey so far and invited students to engage directly with the prototype, exploring functionality, content flow, and interactive features. Structured feedback allowed participants to highlight strengths, identify areas for improvement, and propose refinements. The workshop concluded with collaborative discussions around implementation strategies and evaluation metrics to ensure the e-resource’s practicality, sustainability, and pedagogical effectiveness. A final focus group captured participants’ reflections on the complete co-design process, the perceived quality and relevance of the e-resource, and recommendations for future iterations. Focused ethnographic observation captured how students engaged with the synthesised prototype and collectively negotiated implementation and evaluation considerations during final feedback and planning discussions. The primary outcome of this stage was a finalised, co-designed gamified e-resource ready for implementation in pre-registration health professional curricula.

### Focused ethnography

Focused ethnography was embedded throughout all four AEBCD co-design workshops to examine participation processes, interactional dynamics, and power relations during co-design activities. Focused ethnography is characterised by short-term, intensive engagement within bounded social settings and is well suited to studying specific practices in intervention-oriented research [47]. Unlike traditional ethnography, which typically involves prolonged immersion and broader cultural inquiry, focused ethnography enables in-depth exploration of discrete phenomena within time-limited encounters [36, 53]. In this study, the bounded context comprised four structured co-design workshops involving pre-registration health professional students, with a targeted focus on co-design interactions and decision-making processes. Observational attention was directed towards participation patterns, facilitation dynamics, moments of consensus or tension, and the ways activities and participant-generated artefacts shaped discussion. Operationally, this involved real-time observation of whole-group and small-group activities, including verbal and non-verbal interactions, facilitation strategies, and use of artefacts to support discussion and decision-making. This approach enabled examination not only of what was produced through co-design, but how collaborative decisions unfolded in practice. The focused ethnographic approach aligned with calls for greater transparency in reporting co-design processes, particularly in relation to power sharing, stakeholder influence, and interactional dynamics [54]. Workshops were facilitated by an experienced qualitative researcher (NM) with prior experience in co-design and health professions education, supported by a supervisory team with expertise in qualitative methods, ethnography, and co-design.

Analysis of ethnographic field notes was underpinned by RQI, a structured but flexible approach suited to applied healthcare research where timely findings are required to inform ongoing intervention development [55]. Rapid qualitative approaches support near real-time feedback, stakeholder engagement, and adaptive refinement while maintaining analytic credibility [56, 57]. In this study, RQI enabled iterative reflection between workshops, informed reflexive facilitation, and supported responsive modification of workshop structure and materials. For example, early observations of participant hesitancy and uncertainty in Workshop 1 informed the introduction of structured recaps, optional preparatory materials, and name badges in subsequent workshops, while observations of increasing confidence and self-directed collaboration supported a more facilitative and less directive approach by researchers in Workshops 2 and 3. These iterative refinements were documented and reviewed between workshops to support ongoing adaptation of the co-design process.

### Data collection

Data collection occurred across four co-design workshops, and four post-workshop focus groups and comprised three complementary sources: focused ethnographic field notes, participant-generated artefacts, and audio-recorded focus group discussions. Workshops were facilitated by one lead researcher (NM) and supported by approximately two additional members of the research team (GM, SC, TA, NF). The lead researcher primarily observed whole-group discussions and facilitation processes, while supporting researchers observed small-group activities. Observations were guided by broad areas of interest linked to the study aims, including participation, group dynamics, and engagement with activities. Field notes were recorded during workshops and expanded immediately afterwards. Participant-generated artefacts, including journey maps, sticky notes, dot-voting outputs, ranked topic lists, flipcharts, and prototypes (e.g., storyboards and flowcharts), were collected and photographed (Supplementary 3). Focus groups were conducted immediately after each workshop (approximately 30 min) using a semi-structured topic guide (Supplementary 4), audio-recorded, and transcribed verbatim. Data collection was iterative, with emerging findings informing subsequent workshop facilitation, materials, and prompts.

### Data analysis

Quantitative demographic data were analysed descriptively to contextualise the sample, including gender, age, ethnicity, field, and year of study. Qualitative data comprised focused ethnographic field notes, participant-generated artefacts, and post-workshop focus group transcripts. Focused ethnographic field notes were analysed using RQI to examine co-design dynamics and support iterative workshop refinement. Following each workshop, field notes were reviewed by N.M. and discussed with the supervisory team to identify key patterns in participation, interaction, and engagement. In line with RQI principles, data collection, analysis, and refinement occurred iteratively through cycles of observation, rapid analysis, and adaptation, with emerging findings informing subsequent workshop facilitation, materials, and prompts [55]. Rapid analytic summaries and brief analytic memos documented key observations and resulting modifications between workshops. Focus group data were analysed using reflexive thematic analysis following Braun and Clarke’s (2023) six-step framework [58]. Audio recordings were transcribed verbatim, anonymised, and managed using NVivo (version 15). Analysis was inductive, with coding conducted at semantic and latent levels before being refined into themes capturing patterned meanings across participants’ experiences of co-design. Analysis was led by N.M., with regular team discussions to support reflexive interpretation and analytic rigour. Field notes contextualised focus group accounts, while focus group themes provided explanatory insight into observed workshop dynamics, strengthening the credibility and coherence of the analysis.

### Rigour and reflexivity

Methodological rigour was strengthened through triangulation of focused ethnographic field notes, focus group transcripts, and participant-generated artefacts. Iterative feedback between data collection, analysis, and workshop design enabled emerging findings to inform subsequent facilitation and data generation, supporting analytic credibility and responsiveness [59]. Reflexive thematic analysis was conducted with ongoing team discussion to challenge assumptions and support transparency [58]. Reflexivity was treated as an ongoing process, supported through analytic memo-writing that examined researcher positionality, particularly the dual role of facilitator and observer (NM). This role was managed by prioritising facilitation during workshop activities while supporting researchers (GM, SC, TA, NF) led observations of small-group interactions. Reflexive notes and regular supervisory debriefs were used to distinguish facilitation decisions from observational interpretations and to critically consider potential bias. Additional oversight from the supervisory team and International Observer Panel (IOP) strengthened interpretive rigour and reduced the risk of misrepresentation.

## Results

### Co-design workshops

The results draw on qualitative data from four co-design workshops and accompanying focus groups, triangulated with participant-generated artefacts (Supplementary 3) and field notes from the embedded focused ethnography (Supplementary 5). Demographic data are reported descriptively to contextualise the sample (Table 4). Analysis focused on students’ experiences of the co-design process, rather than on evaluation of the final e-resource. A total of 15 pre-registration health professional students participated in the co-design workshops and focus groups. Participants represented nursing and pharmacy disciplines, with diversity in year of study, gender, ethnicity and age shown in Table 4. The sample was predominantly female (*n* = 11) and white (*n* = 12), with smaller representation from other (*n* = 3) backgrounds. Most participants were aged 18–21 (*n* = 12). Participants were primarily from pharmacy programmes (*n* = 10), followed by nursing students (*n* = 5). The majority were in their first year of study (*n* = 10).

**Table 4:** Co-design Participant Demographics *

	*n*	%
Age Group		
18–21	12	80.0
22–25	1	6.7
31–40	1	6.7
46–50	1	6.7
Gender		
Male	4	26.7
Female	11	73.3
Ethnicity		
White	12	80.0
Other	3	20.0
Field of Study		
Nursing	5	33.3
Pharmacy	10	66.6
Year of Study		
Year One	10	66.6
Year Two	2	13.3
Year Three	2	13.3
Year Four	1	6.7

*Percentages may not total 100% due to rounding

**Fig. 5:**
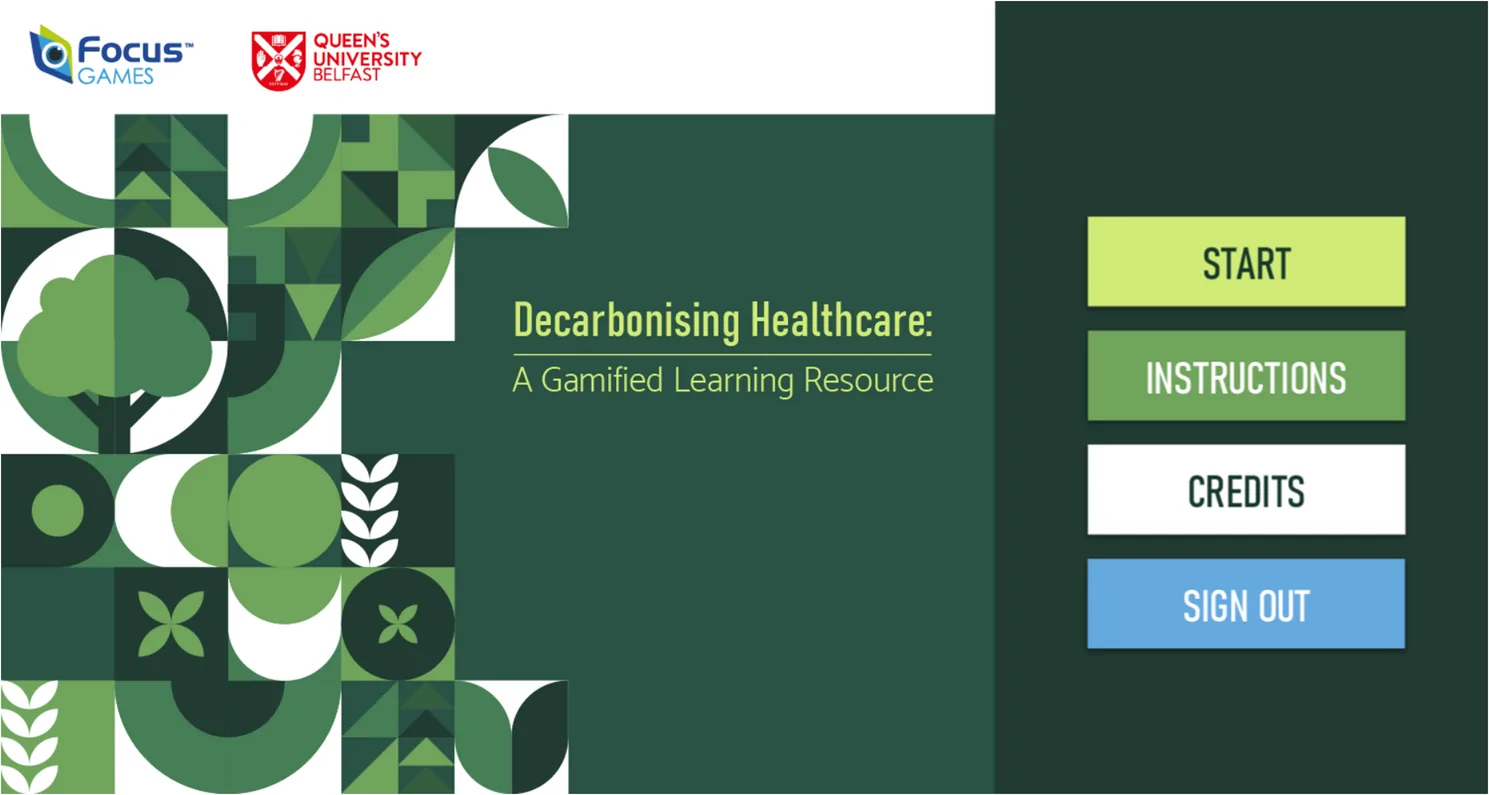
Screenshot of E-resource

The co-design workshops resulted in the content for a four-module e- resource on healthcare decarbonisation, reflecting the priorities and perspectives of pre-registration health professional students (Fig. 5). The first module, *Introduction: Climate Change*,* Health & Sustainability*, focused on the foundational science of climate change, the health impacts of environmental change, core sustainability concepts, and the emerging role of healthcare professionals in low-carbon, equitable care. This structure reflected early student priorities to *“show reality and urgency of global warming”* and highlight that *“vulnerable [populations are] most impacted but least responsible”* (Workshop 2, pg.1). The second module, *Core Concepts of Healthcare Decarbonisation*, addressed sources of emissions within healthcare, high-impact clinical areas, sustainable models of care, and real-world case studies to contextualise learning. This responded to students’ requests to *“relate to health and healthcare”* and include *“pie chart of emissions in healthcare”* and *“context specific examples”* (Workshop 2, pg.1). Module three, *Decarbonisation in Practice: Knowledge to Action*, emphasised practical low-carbon clinical actions, interprofessional collaboration, and scenario-based application to develop confidence in implementing sustainable practices. This reflected students’ priorities such as *“individual actions on how to decarbonise”*, *“barriers paired with practical examples for change”*, and *“real world examples of change”* (Workshop 2, pg.2). The final module, *Tools*,* Frameworks and Policy*, introduced participants to professional standards, measurement tools, sustainable quality improvement principles, and international resources to support systemic change. This aligned with student requests to *“finish on a positive note”*, *“practical actions and solutions”*, and *“signposting to other resources”* (Workshop 2, pg.3). Students intentionally structured the e-resource to move from global concepts to actionable tools, reflecting their desire for agency and application in practice.

Participants prioritised digital and gamified features that would maximise engagement while maintaining a serious and evidence-based tone. Proposed design elements included *“gamification – quizzes*,* leaderboard*,* certificate at the end”*, *“navigate a scenario – make decisions based on a healthcare scenario”*, and *“progress marker”* (Workshop 3, pg.1). Students also emphasised usability, advocating for *“simple plain font”*, *“not too much information – organised”*, and *“easy transitions between slides”* (Workshop 3, pg.1). Students emphasised the importance of concise, accessible modules that could be completed across devices without imposing excessive time burdens, “*simplicity” and “be clear how to navigate”* (Workshop 3, pg,1).

**Fig. 6:**
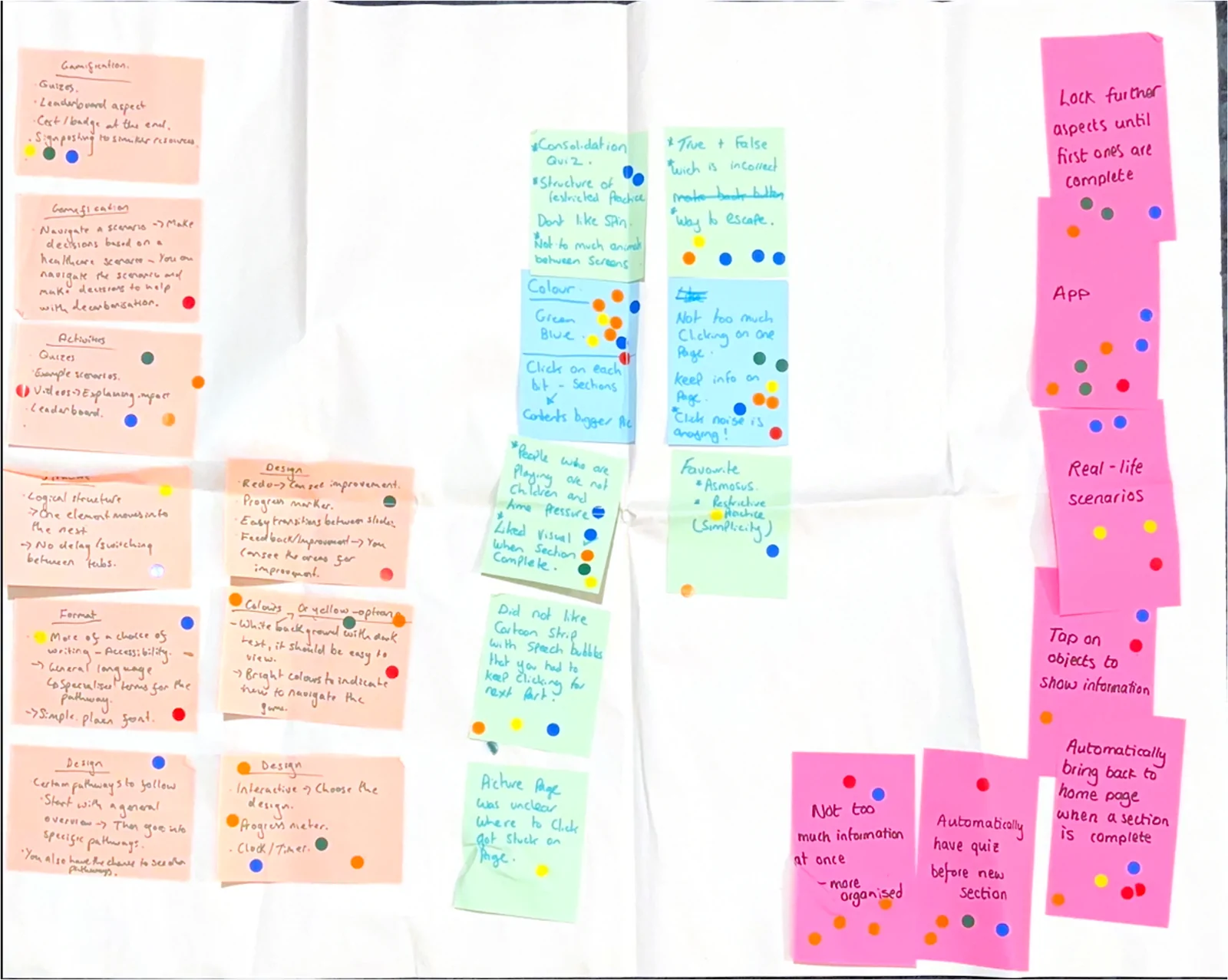
Workshop 3 pg 1 Artefact

**Fig. 7:**
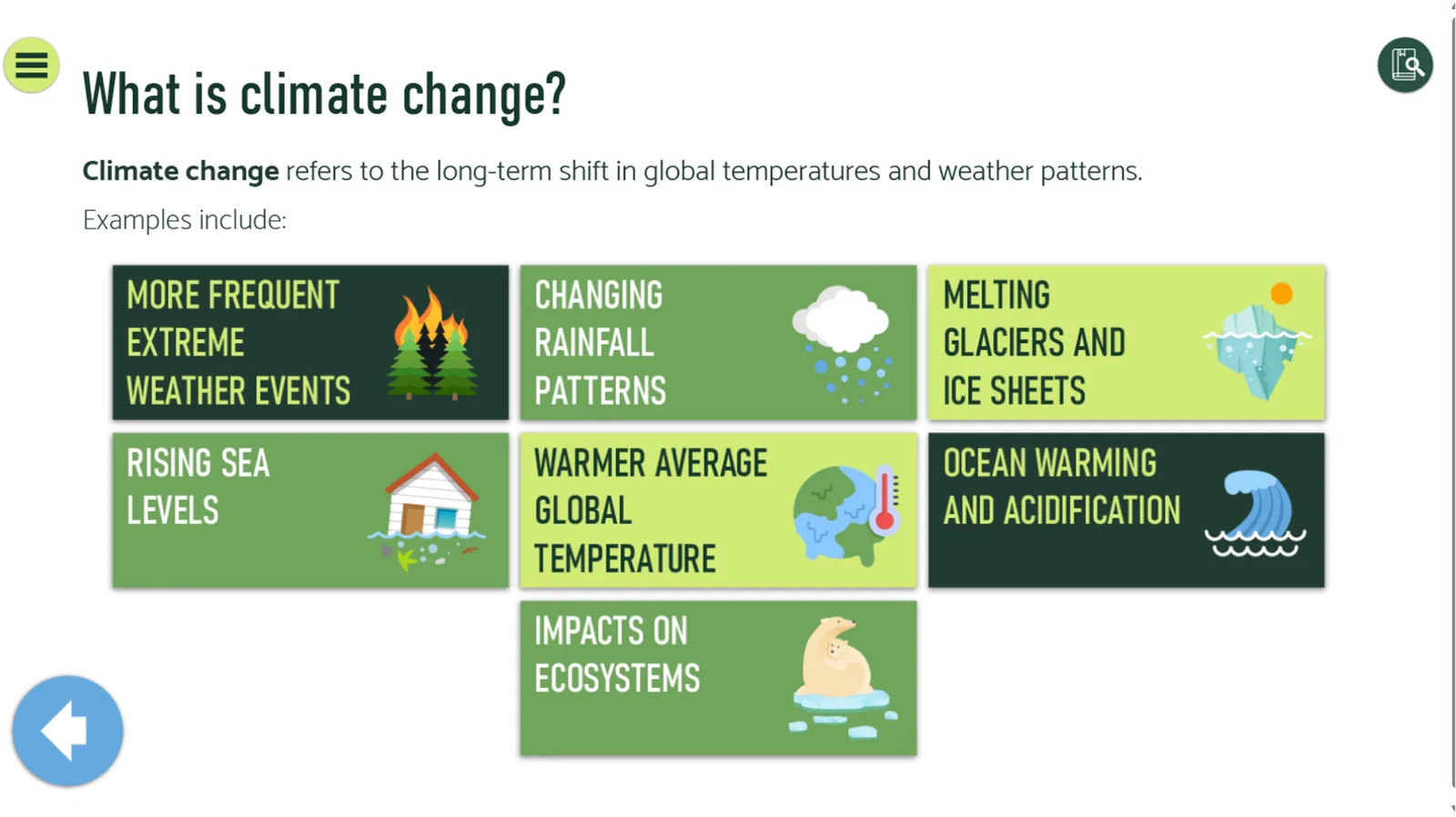
Screenshot of E-resource

Artefact data further illustrated how participants negotiated content scope, pedagogical clarity, and feasibility (Fig. 6). Early experience mapping highlighted sustainability concerns such as *“PPE wastage”*, *“food waste in hospitals”*, and *“medication packaging waste”* (Workshop 1, pg.1), alongside emotional responses including *“helpless”*, *“frustrated”*, and *“sometimes feel my small effort won’t make a difference”* (Workshop 1, pg.2–3). These findings directly informed the inclusion of practical action-oriented content and reflective activities within the modules. Overall, the artefacts demonstrate a structured and iterative co-design process, with student input directly shaping both the content and format of the final e-resource (Fig. 7).

### Post-workshop focus groups

Three overarching themes were identified across the four co-design workshops and associated focus groups: (1) Agency and Confidence in Action, (2) Interdisciplinary Collaboration and Shared Purpose, and (3) Structured Facilitation and Tangible Outputs. Themes were generated through iterative analysis of focus group transcripts, workshop artefacts (e.g., flipcharts, sticky notes, dot-voting outputs, and digital thought clouds), and reflexive ethnographic field notes, enabling patterns to be examined across the programme rather than within isolated sessions.

#### Theme 1: agency and confidence in action

Students described the co-design process as more accessible and less intimidating than anticipated, despite limited prior experience of research, curriculum development, or co-design. Rather than feeling like passive consultees, participants reported developing confidence, voice, and a sense of agency over time. Initial uncertainty was common, particularly around contributing ideas without perceived expertise: “The first workshop I was a bit apprehensive because I had never been involved in this sort of thing before…” (P1 FG3). However, repeated engagement, supportive facilitation, and clear workshop structure increased participants’ confidence: “…once you have a bit of background knowledge and see how it works, you get a bit more confident in getting your ideas out there.” (P1 FG3).

**Fig. 8:**
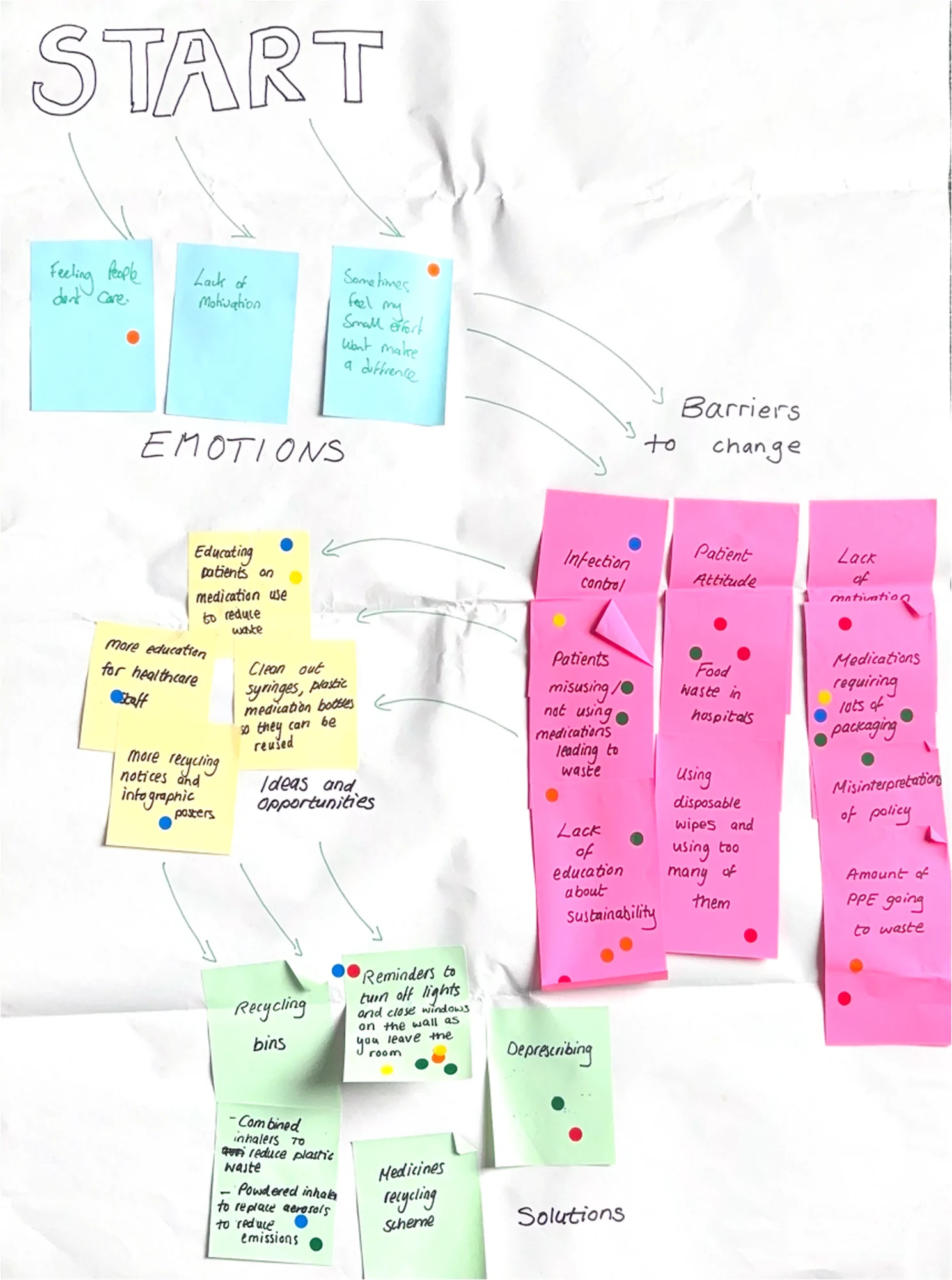
Workshop 1 pg 3 Artefact

This progression was reflected in focused ethnographic field notes, which documented a shift from “early verbal contributions [that] were hesitant, often framed as questions” (FNWS1, Supplementary 5) to “sustained, animated discussion… with minimal facilitator input” (FNWS3, Supplementary 5). By Workshop 3, students independently negotiated priorities, structured group tasks, and defended design decisions during feedback sessions. Artefact data demonstrated a similar transition. Early experience maps captured feelings of limited influence, including “sometimes feel my small effort won’t make a difference” and “helpless” (WS1, pg.3), whereas later artefacts prioritised practical solutions such as “deprescribing,” “medication recycling scheme,” and “combined inhalers to reduce plastic waste” (WS1, pg.3) (Fig. 8). By Workshop 2, students explicitly prioritised “individual actions on how to decarbonise” and “barriers paired with practical examples for change” (WS2, pg.2), indicating a shift from problem identification towards action-oriented thinking.

Participants consistently emphasised the importance of feeling listened to and valued, regardless of year of study or professional background. One first-year student reflected, “Even from just sitting here, I’m only a first year and I’m able to talk with people who are more experienced and share them ideas” (P3 FG1). Students described the workshops as psychologically safe spaces where contributions were welcomed without judgement. This was supported by field notes documenting “increased eye contact, overlapping speech, and spontaneous affirmation… suggesting emerging psychological safety” (FNWS1, Supplementary 5). Observations also captured instances where ideas proposed by junior students were subsequently incorporated into the evolving e-resource, reinforcing perceptions that contributions had tangible influence.

Beyond the workshops, students reported increased awareness of sustainability and healthcare decarbonisation in both professional and personal contexts. Participants described becoming more attentive to opportunities for change in practice: “You definitely would be more aware… you start to kind of see more opportunities for decarbonisation” (P6 FG3). This was reflected in artefacts identifying practical interventions such as “sensor taps,” “reusable PPE kits,” and “efficient and intuitive storage to prevent wasting resources” (WS1, pg.2). Increased awareness persisted through to the final focus group, where one participant reflected that “even since I started this process… I would have been actively thinking… what could be different?” (P1 FG4). Together, these findings suggest that participation in the co-design process fostered confidence, ownership, and greater readiness to identify and act on sustainability opportunities.

#### Theme 2: interdisciplinary collaboration and shared purpose

Interdisciplinary interaction emerged as a defining feature of the co-design process, shaping both engagement with healthcare decarbonisation and perceptions of the e-resource. Students consistently valued learning alongside peers from another health profession, describing how exposure to different perspectives broadened their understanding of sustainability in practice. One participant noted, “I just find it interesting to hear about other people’s perspectives on healthcare in general and hearing from all the different backgrounds” (P10 FG3). Rather than creating conflict, disciplinary differences were viewed as complementary, with participants reflecting that “…we weren’t arguing about the fact we’ve got different views… I like that you put everything on the board and you see what everybody thinks or likes” (P1 FG3).

**Fig. 9:**
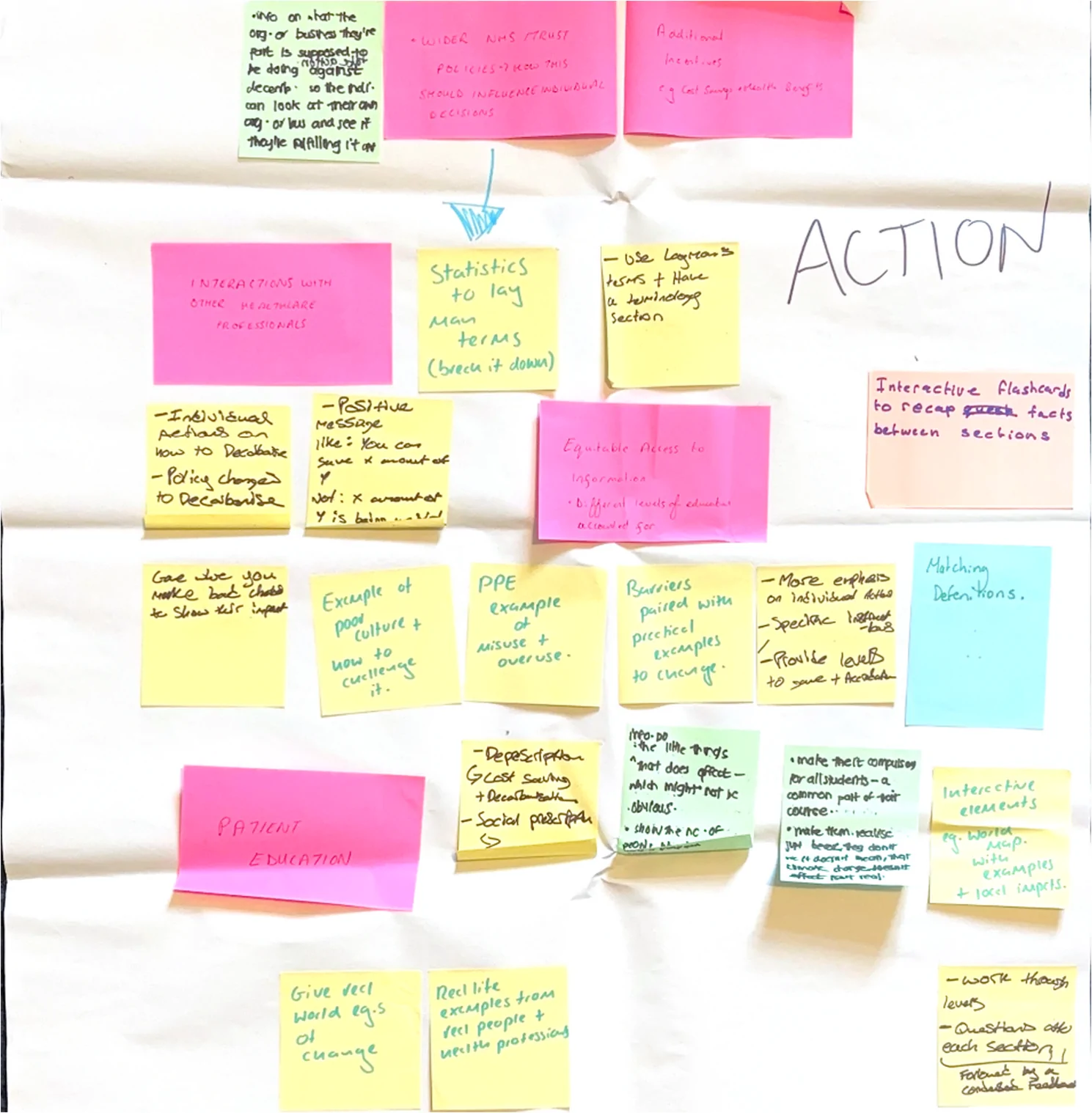
Workshop 2 pg 2 Artefact

Focused ethnographic observations supported these accounts, documenting how students actively built on one another’s ideas and negotiated differences constructively. Field notes described “disciplinary identity invoked as a resource rather than a boundary… mixed-discipline groupings led to visible moments of comparison and synthesis” (FNWS2, Supplementary 5). This was reflected in participant-generated artefacts, where nursing and pharmacy students collaboratively clustered content into shared modules (e.g., Global, Health, Action, and Signposting) and proposed integrating topics such as “case studies of patients,” “PPE misuse examples,” “social prescribing,” and “policy changes to decarbonise” within the same learning pathway (WS2, pg.1–3; Fig. 9). These outputs demonstrated how interdisciplinary discussion shaped the breadth and integration of content, reinforcing healthcare decarbonisation as a collective rather than profession-specific responsibility.

Small-group working was identified as a key mechanism supporting participation and collaboration. Students reported that pairs and triads reduced anxiety and encouraged idea sharing: “…in a bigger group you might be kind of more nervous to voice your ideas, so I think that facilitated the sharing of ideas a bit more” (P6 FG3). Field notes documented that students quickly self-organised, with “students self-assigned roles… and timekeeping emerged organically within groups” (FNWS2, Supplementary 5). Researchers observed disciplinary expertise being shared and negotiated, with pharmacy students often contributing medication-related sustainability perspectives, while nursing students highlighted practice-based opportunities such as reducing waste and resource use. This exchange fostered mutual learning and enabled participants to build on one another’s expertise.

Across focus groups, students described a strong sense of shared purpose, recognising sustainability as a challenge requiring collective action. One participant reflected that “…working in groups increases the threshold for how many of these ideas you can come up with, if we had tried to do this individually, we would have definitely not come up with the same thing. Not at the same quality” (P5 FG2). Similarly, another noted that participation highlighted how people often share ideas about sustainability but require encouragement and opportunities to act on them (P2 FG1). Ethnographic observations documented high levels of engagement, with students collectively negotiating learning priorities and e-resource design decisions, while final workshop reflections highlighted increased appreciation of other professional roles, with participants noting that the resource helped them understand “what impact other people in the MDT have… from another person’s perspective” (P5 FG4). Together, these findings suggest that interdisciplinary collaboration fostered inclusivity, shared ownership, and a collective commitment to sustainable healthcare practice.

#### Theme 3: structured facilitation and tangible outputs

Students attributed their positive experiences of co-design to the clarity, structure, and materiality of the workshops, which supported sustained engagement and ownership of outcomes. Facilitation was frequently described as enabling participation and maintaining direction: “The facilitation helped us stay directed and clarify what you mean through the actual discussion” (P3 FG2). Advance emails, session outlines, preparatory materials, and workshop recaps were also valued, helping students feel informed and confident in contributing. As one participant noted, “…you can see much more, much broader range reflected in some of the papers you send on and even with some of the like online games and apps you can kind of explore the different options. That’s nice” (P6 FG3). Focused ethnographic field notes supported these accounts, documenting that students who engaged with preparatory materials participated more readily and required less facilitation, with discussions becoming “flowing and more natural” over time (FNWS2, Supplementary 5). Structured recaps also reinforced continuity and responsiveness, with students “immediately recognised artefacts from the previous session… indicating continuity and ownership” (FNWS2, Supplementary 5), while visible changes between workshops were perceived as making sessions feel “more professional and easier to settle” (FNWS3, Supplementary 5).

**Fig. 10:**
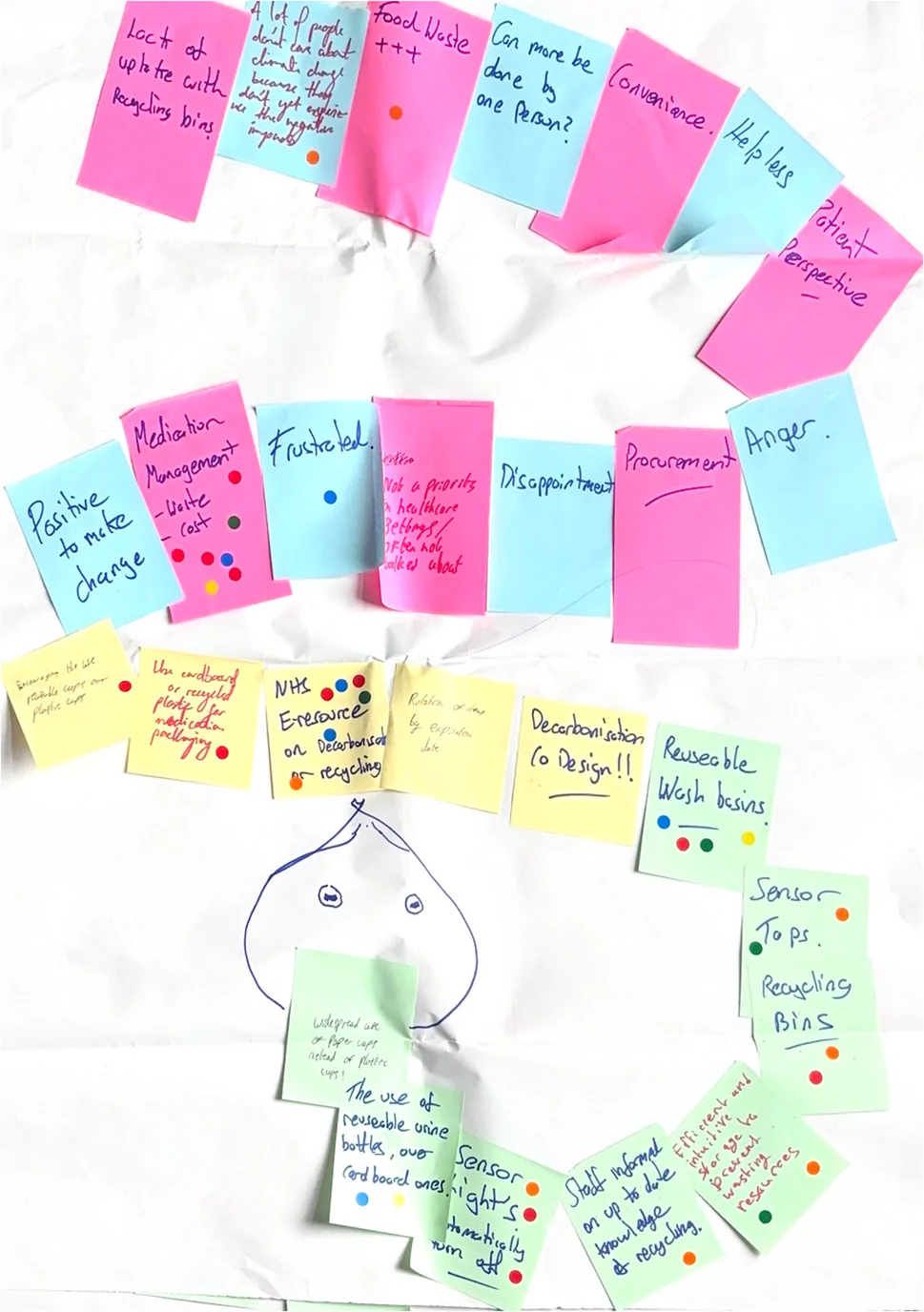
Workshop 1 pg 2

Physical and visual artefacts played a central role in structuring discussion and supporting collaborative decision-making. Participants described sticky notes, flipcharts, dot voting, and thought clouds as helping them organise ideas and make contributions visible: “…it helps you organise your thoughts and not forget things…” (P10 FG3). Artefacts enabled participants to identify barriers, solutions, and priorities, while supporting negotiation and consensus-building around module content, learning objectives, and design features. For example, colour-coded journey maps captured emotional responses, barriers, ideas, and solutions during Workshop 1, while Workshop 2 artefacts clustered content into shared modules (Global, Health, Action, and Signposting) (WS1–2, pg.1–3). Ethnographic observations documented students “pointing across groups to align ideas” and “repositioning content through discussion” (FNWS2, Supplementary 5), illustrating how artefacts scaffolded collective reasoning. Similarly, dot-voting exercises enabled transparent prioritisation of challenges and preferred design features (Fig. 10). Students also valued interactive elements within the prototype, reporting that having to “keep clicking on things… made you pay attention rather than just next, next, next” (P2 FG4).

Participants placed particular value on seeing their contributions translated into tangible outputs across workshops. One student observed, “…you’ve actually listened to our ideas and then done that in the next workshop” (P1 FG3), while another reflected, “I feel quite proud to be part of something actually” (P1 FG3). Priorities identified in early workshops reappeared in later learning objectives, interactive scenarios, quizzes, and case studies (WS3, pg.1). Field notes documented participants “explicitly linking design decisions back to earlier priorities” (FNWS3, Supplementary 5) and increasingly describing the e-resource as something they had “made rather than contributed to” (FNWS3, Supplementary 5). This visible integration of student input strengthened ownership, legitimacy, and investment in the final resource, with participants also offering implementation suggestions such as delivering the resource “close to placement so it’s on a person’s mind” (P4 FG4), demonstrating engagement with its future application beyond the co-design process.

## Discussion

This study primarily demonstrates the value of integrating AEBCD with focused ethnography and rapid qualitative approaches to enhance the transparency and responsiveness of co-design processes. It illustrates how this approach can be applied to develop a learner-centred e-resource on healthcare decarbonisation. Rather than evaluating educational effectiveness, the contribution of this work lies in demonstrating methodological and pedagogical value of co-design for developing sustainability education. By documenting both the outputs and the lived experience of participation, this study responds to calls for greater methodological transparency and reflexivity in co-design research [28, 29, 60]. The findings suggest that AEBCD, when combined with ethnographic insight, can support not only e-resource development but also student engagement, agency, emerging professional identity and interprofessional collaboration. However, these outcomes should be interpreted considering the co-design context, where structured facilitation, group-based interaction, and the presence of researchers may have shaped how participants expressed ideas and engaged with the process. Formal evaluation of the acceptability of the co-designed e-resource is required and will be reported separately [61].

The use of AEBCD was particularly well suited to the challenges of healthcare decarbonisation education. Sustainability is frequently experienced by students as an area of limited understanding and preparedness [62–64]. AEBCD enabled rapid yet meaningful engagement with these complexities by foregrounding students’ lived educational experiences and using narrative-driven trigger materials to support collaboration. The co-design process shifted sustainability away from a purely technical or moral framing, creating space for students to consider its relevance to their curriculum, professional responsibilities, and agency. This aligns with growing recognition that sustainability education requires approaches that address values, identity, and behavioural capability, not knowledge alone [65–67]. The findings indicate that AEBCD can help legitimise student perspectives on complex topics and support ownership of learning resources.

Embedding focused ethnography within the AEBCD process added critical analytic depth by illuminating how students experienced participation, rather than focusing solely on what they produced. Ethnographic observation captured interactional dynamics, moments of uncertainty, and shifts in confidence that may not be fully visible through artefacts or focus group data alone [68, 69]. This enabled interpretation of co-design not simply as a method for e-resource development, but as a pedagogical process with developmental consequences [70]. Focused ethnography functioned as an analytic lens that contextualised artefact production and thematic findings, revealing how facilitation strategies, group composition, and material tools shaped engagement [71]. This also highlighted that participation was not neutral; dominant voices, prior experiences, and disciplinary perspectives may have influenced which ideas were amplified, negotiated, or deprioritised within group settings. In doing so, the study moves beyond instrumental accounts of co-design towards a more reflexive understanding.

Positioned within the wider co-design literature, this study builds on established applications of EBCD and AEBCD by extending their use into sustainability and decarbonisation education, an area where co-design remains underexplored [39]. While sharing methodological features with prior AEBCD studies, such as iterative workshops and artefact development, the educational focus and participant group distinguish this work conceptually and methodologically. Recent work highlights the value of involving students as co-designers in health professions education, demonstrating how participatory approaches can shape learning resources and pedagogical processes [22, 72]. Specific applications in sustainability and climate-health education further support the relevance of co-design in this domain, including co-designed interventions addressing sustainable development goals and planetary health competencies [73–75]. By applying AEBCD to climate-health education, the study responds to calls for innovative pedagogies capable of addressing the complexity, urgency, and ethical dimensions of healthcare decarbonisation. However, the extent to which these findings can be generalised is shaped by the specific composition of the participant group and the structured nature of the co-design process, which may not fully capture the diversity of perspectives across all health professions. *Transferability is likely to depend on factors such as institutional support*,* availability of stakeholder engagement opportunities*,* facilitation expertise*,* and the ability to accommodate iterative refinement within educational development timelines. The approach could be adapted for different health professions*,* learner groups*,* and cultural contexts by tailoring trigger materials*,* workshop activities*,* and participant composition to local priorities and educational needs*,* while retaining the core principles of collaborative decision-making*,* iterative feedback*,* and reflexive inquiry.*

Students described healthcare decarbonisation as a shared responsibility, reinforcing the potential value of interprofessional sustainability education. This was primarily reflected through nursing and pharmacy perspectives, which may have influenced the emphasis placed on particular areas such as medication-related sustainability and practice-level interventions. While the specific e-resource developed here will require contextual adaptation, the combined co-design and focused ethnography approach may be transferable to other educational settings with similar participant demographics. *Although this study focused on healthcare decarbonisation*,* the methodological approach may also be applicable to the development of education addressing broader planetary health and sustainable healthcare challenges*,* including resource stewardship*,* waste reduction*,* and overmedicalisation. The urgency of the planetary health crisis requires timely action to prepare healthcare students and professionals for sustainable practice. However*,* this urgency does not remove the need for educational resources to be relevant*,* accessible*,* and aligned with learners’ needs. Rather*,* it reinforces the value of pragmatic co-design approaches that can support timely resource development while maintaining learner engagement and contextual relevance. The accelerated process used in this study may offer a feasible approach for developing sustainability-focused learning resources within educational timelines. Future work could adapt the trigger materials*,* workshop structure*,* learner-derived priorities*,* and design principles generated through this study to support more rapid development of educational resources across broader planetary health topics*,* without necessarily repeating the full co-design process in every setting.*

This e-resource aligns with broader calls for whole-system approaches to sustainability education that cut across professional silos, emphasising collective responsibility and preparing future health professionals to respond to climate-related health challenges [76–80].

### Strengths and limitations

A key strength of this study is the use of AEBCD to develop an e-resource addressing healthcare decarbonisation, an area where empirically grounded educational interventions remain limited. The interdisciplinary co-design approach supported meaningful engagement with pre-registration health professional students and enabled the development of an e-resource that reflects learner priorities, contextual realities, and perceived educational needs. The integration of iterative workshops and post-workshop focus groups enhanced the depth of insight into the co-design process and supported reflexive refinement across workshops. This study has several limitations related to sample composition. Although recruitment targeted a broad range of health professions, participation was limited to nursing and pharmacy students, with a predominance of pharmacy participants and first/second year students. As a result, the co-design process is more accurately characterised as a nursing–pharmacy collaboration rather than a fully interdisciplinary. The sample was also largely female and of similar ethnic background, which may have influenced results. These characteristics likely shaped both the content and emphasis of the co-designed outputs. *As participation was voluntary*,* self-selection bias is possible*,* and participants may have had a greater interest in sustainability than the wider student population.* In addition, the workshops were conducted at a single institution, limiting broader contextual generalisability.

### Lessons learned

This study highlights several methodological lessons for co-design in health professions education. Combining structured co-design frameworks with focused ethnography and rapid qualitative analysis enabled both in-depth understanding and real-time adaptation of the process. Ensuring visible integration of student input, alongside the use of artefact-based activities and small-group work, supported engagement, ownership, and collaborative decision-making. These considerations may support the transferability and operationalisation of co-design approaches in similar contexts. Methodologically, the integration of AEBCD, focused ethnography, and RQI demonstrated how participatory design can be complemented by process-oriented inquiry and iterative analysis, enabling researchers to examine not only what is co-designed, but how design decisions emerge, evolve, and are negotiated over time. This may be particularly valuable in health professions education and educational innovation where interventions are developed alongside ongoing stakeholder engagement and refinement. This approach may also be transferable to continuing professional development (CPD) and postgraduate education, supporting scalable and inclusive climate education across diverse healthcare roles and career stages.

## Conclusion

This study describes the co-design of an e-resource on healthcare decarbonisation using an AEBCD approach with an embedded focused ethnography. While further research is required to evaluate the e-resource’s acceptability, these findings suggest that this approach is well suited to engaging pre-registration health professional students with the complexity, uncertainty, and practical realities of healthcare decarbonisation and could be applied to other topics in health professions education. The co-design process supported shared understanding, interdisciplinary collaboration, and the development of student agency by positioning learners as legitimate contributors to educational innovation. Importantly, the integration of focused ethnography enhanced transparency around how design decisions emerged, offering a reflexive and transferable methodological approach for developing future educational interventions in health professions education.

## Supplementary Information

Supplementary Material 1.

## Data Availability

The anonymized transcripts used and/or analysed during the current study are available from the corresponding author on reasonable request.

## Abbreviations

AEBCD: Accelerated Experience–Based Co–Design
EBCD: Experience–Based Co–Design
FE: Focused Ethnography
FG: Focus Group
FN: Field Notes
GHG: Greenhouse Gas
IOP: International Observer Panel
MDT: Multidisciplinary Team
PPE: Personal Protective Equipment
RQI: Rapid Qualitative Inquiry
WS: Workshop
