# Supplementary material for: Student participation in accelerated experience-based co-design for healthcare decarbonisation education: a focused ethnographic study
Source: BMC Med Educ. 2026 Jul 25;26:1687. doi: 10.1186/s12909-026-10015-1 (PMC13685220; doi:10.1186/s12909-026-10015-1)
Supplement: Supplementary file 1 [file 12909_2026_10015_MOESM1_ESM.docx]

Supplementary 1: GRIPP2 long form checklist

| **Section and topic** | **Item** | **Reported on page No** |
| --- | --- | --- |
| Section 1: Abstract of paper | | |
| 1a: Aim | Report the aim of the study | 2 |
| 1b: Methods | Describe the methods used by which patients and the public were involved | 2 |
| 1c: Results | Report the impacts and outcomes of PPI in the study | 2 |
| 1d:Conclusions | Summarise the main conclusions of the study | 2 |
| 1e: Keywords | Include PPI, “patient and public involvement,” or alternative terms as keywords | 2 |
| Section 2: Background to paper | | |
| 2a: Definition | Report the definition of PPI used in the study and how it links to comparable studies | 3-5 |
| 2b: Theoretical underpinnings | Report the theoretical rationale and any theoretical influences relating to PPI in the study | 3-5 |
| 2c: Concepts and theory development | Report any conceptual or theoretical models, or influences, used in the study | 3-5 |
| Section 3: Aims of paper | | |
| 3: Aim | Report the aim of the study | 5 |
| Section 4: Methods of paper | | |
| 4a: Design | Provide a clear description of methods by which patients and the public were involved | 5-21 |
| 4b: People involved | Provide a description of patients, carers, and the public involved with the PPI activity in the study | 7&8 |
| 4c: Stages of involvement | Report on how PPI is used at different stages of the study | 5-17 |
| 4d: Level or nature of involvement | Report the level or nature of PPI used at various stages of the study | 5-17 |
| Section 5: Capture or measurement of PPI impact | | |
| 5a: Qualitative evidence of impact | If applicable, report the methods used to qualitatively explore the impact of PPI in the study | 5-17 |
| 5b: Quantitative evidence of impact | If applicable, report the methods used to quantitatively measure or assess the impact of PPI | N/A |
| 5c: Robustness of measure | If applicable, report the rigour of the method used to capture or measure the impact of PPI | 17 |
| Section 6: Economic assessment | | |
| 6: Economic assessment | If applicable, report the method used for an economic assessment of PPI | N/A |
| Section 7: Study results | | |
| 7a: Outcomes of PPI | Report the results of PPI in the study, including both positive and negative outcomes | 17-27 |
| 7b: Impacts of PPI | Report the positive and negative impacts that PPI has had on the research, the individuals involved (including patients and researchers), and wider impacts | 17-27 |
| 7c: Context of PPI | Report the influence of any contextual factors that enabled or hindered the process or impact of PPI | 17-27 |
| 7d: Process of PPI | Report the influence of any process factors, that enabled or hindered the impact of PPI | 17-27 |
| 7ei: Theory development | Report any conceptual or theoretical development in PPI that have emerged | N/A |
| 7eii: Theory development | Report evaluation of theoretical models, if any | N/A |
| 7f: Measurement | If applicable, report all aspects of instrument development and testing (eg, validity, reliability, feasibility, acceptability, responsiveness, interpretability, appropriateness, precision) | N/A |
| 7 g: Economic assessment | Report any information on the costs or benefit of PPI | N/A |
| Section 8: Discussion and conclusions | | |
| 8a: Outcomes | Comment on how PPI influenced the study overall. Describe positive and negative effects | 28-32 |
| 8b: Impacts | Comment on the different impacts of PPI identified in this study and how they contribute to new knowledge | 28-32 |
| 8c: Definition | Comment on the definition of PPI used (reported in the Background section) and whether or not you would suggest any changes | 28-32 |
| 8d: Theoretical underpinnings | Comment on any way your study adds to the theoretical development of PPI | 28-32 |
| 8e: Context | Comment on how context factors influenced PPI in the study | 28-32 |
| 8f: Process | Comment on how process factors influenced PPI in the study | 28-32 |
| 8 g: Measurement and capture of PPI impact | If applicable, comment on how well PPI impact was evaluated or measured in the study | N/A |
| 8 h: Economic assessment | If applicable, discuss any aspects of the economic cost or benefit of PPI, particularly any suggestions for future economic modelling. | N/A |
| 8i: Reflections/critical perspective | Comment critically on the study, reflecting on the things that went well and those that did not, so that others can learn from this study | 28-32 |

Supplementary 2 – Trigger Materials Table

| **Vignette** | **Source** | **Format** | **Content focus** |
| --- | --- | --- | --- |
| “Before this module, I thought sustainability was something for environmental scientists — not nurses. But now, I can’t unsee how every syringe, every PPE item, every light left on has an impact. It’s made me realise that sustainable care *is* safe care — it’s part of my professional responsibility, not an optional extra.”*– Nursing student, UK* | McLaughlin-Borlace et al. 2026 | Vignette | Theme 1: Building a Decarbonisation Mindset |
| “I used to feel anxious every time I thought about climate change — like it was too big for me to do anything about. But hearing examples of small, practical things healthcare workers are already doing gave me hope. Now I see that my concern can actually drive change, not freeze me.”*– Midwifery student, Türkiye* | McLaughlin-Borlace et al. 2026 | Vignette | Theme 2: From Concern to Constructive Engagement |
| “After the session, I started paying attention to what we throw away in theatre. I realised we open way more single-use items than we use. I talked to my mentor about it — we changed how we set up the tray. It’s small, but it made me feel like I can actually do something real.*– Medical student, Sweden* | McLaughlin-Borlace et al. 2026 | Vignette | Theme 3: Translating Awareness into Practice |
| “What really changed things for me was seeing other students and clinicians talk about sustainability with passion — not as a lecture, but as something we can lead on. It made me think: maybe I can be that person too. I don’t have to wait until I’m senior to start.”*– Nursing student, Canada* | McLaughlin-Borlace et al. 2026 | Vignette | Theme 4: Becoming Low-Carbon Change Agents |
| “On placement, I saw how much waste we create. I wanted to say something — but it didn’t feel like my place. I didn’t want to seem difficult or naive. It’s hard when sustainability feels important to you, but the culture doesn’t support it.” *– Nursing student, Spain* | McLaughlin-Borlace et al. 2026 | Vignette | Theme 5: Barriers to Decarbonisation Action |
| **Resources** |  |  |  |
| “Climate Change Explained” | UK Met Office (YouTube: <https://www.youtube.com/watch?v=d9MYUUt8cK0>) | Short film | Fundamental climate science and anthropogenic drivers |
| “Climate Change 2023: Synthesis Report Trailer” | IPCC (2023) (YouTube: <https://www.youtube.com/watch?v=T9CeECpxtx8>) | Short film | Global climate impacts, mitigation and adaptation imperatives |
| “Climate Change: Natural Carbon Cycle” | Ludwig Maximilian University (LMU) (YouTube: <https://www.youtube.com/watch?v=BRyIIzfI1u4>) | Short film | Carbon cycle processes and human perturbation |
| “The Carbon Cost of Healthcare” | The Lancet (2023) (YouTube: <https://www.youtube.com/watch?v=Nap2p4EeUQo>) | Short film | Healthcare sector emissions and system-level decarbonisation |
| “Healthcare Sustainability” | Royal College of Physicians (2018) (YouTube: <https://www.youtube.com/watch?v=g3xTfNEwfyM>) | Short film | Clinical practice, professional responsibility, and sustainability |

Supplementary 3 – Workshop 1-3 Artefacts (complete)

| **Workshop / pg** | **Artefact** |
| --- | --- |
| 1 pg 1 |  |
| 1 pg 2 |  |
| 1 pg 3 |  |
| 2 pg 1 |  |
| 2 pg 2 |  |
| 2 pg3 |  |
| 3 pg 1 |  |

Supplementary 4- Focus Group Interview Guide

**Focus-Group Interview Guide – Co-Design**

**Title of Project**: Co-Design of a Gamified E-Resource about Healthcare Decarbonisation.

**MHLS Ethics approval number:** MHLS 25_96

**Welcome and Introductions – 5 minutes**

**(All focus-group interviews to be moderated by two members of the research team)**

**Explain purpose-** **5 minutes**

The purpose of this focus-group is to explore the student perspectives and experiences of the co-design process. There are a few areas I wish to discuss with you about your experience. If any participant wishes to stop or withdraw from the interview at any time, please let me know and the interview will be stopped.

**Focus Group Interview Guide** **– 40 minutes**

- What has your experience of the co-design workshops been like?
- What worked well about the co-design process?
- Tell us what did not work so well?
- What do you think you’ve learned from the co-design workshops?
- What suggestions would you have in designing future co-design workshops?
- What would you like to see happen next?
- Is there anything else that you would like to add that we have not discussed?

**Thanks and Close – 5 minutes**

Supplementary 5 – Researcher Field Notes

**Focused Ethnographic Field Notes: Workshop 1 (FNWS1) – Entering the Co-Design Space**

**Analytic focus:** Initial engagement, psychological safety, early sense-making

At the start of Workshop 1, students entered the room cautiously, with limited informal interaction across tables. Several participants positioned themselves at the edges of seating arrangements, and early verbal contributions were hesitant, often framed as questions or prefaced with uncertainty (e.g., “I’m not sure if this is right…”). This initial tentativeness appeared particularly pronounced among first-year students, suggesting sensitivity to perceived hierarchies of knowledge and experience.

As trigger materials were introduced, including vignettes and short films, the atmosphere shifted. Students leaned forward during the presentations and began making notes independently, indicating cognitive engagement prior to verbal participation. The thought-cloud exercise functioned as a low-risk entry point: contributions increased rapidly once students realised that short words or phrases were acceptable, and emotional terms (e.g., *“frustrated,” “helpless,” “angry”*) appeared frequently. This marked the first visible collective acknowledgement of affective responses to healthcare decarbonisation.

During the storytelling and experience journey mapping activity, the use of colour-coded sticky notes (blue for emotions, red for barriers, yellow for ideas, green for solutions) appeared to legitimise emotional expression alongside practical concerns. Students frequently pointed to notes while speaking, using artefacts as mediating objects rather than speaking directly “from themselves.” This reduced hesitation and supported more confident contributions. There was recorded increased eye contact, overlapping speech, and spontaneous affirmation (e.g., nodding, verbal agreement), suggesting emerging psychological safety.

Dot voting acted as a critical transition point from expression to prioritisation. Observationally, students debated vote placement openly, often negotiating aloud and justifying choices to peers. This marked an early shift from individual reflection to collective decision-making. The workshop concluded with visibly higher energy levels than at entry, and the post-workshop focus group reflected a tone of cautious optimism, aligning with early expressions of emerging agency (Theme 1).

**Focused Ethnographic Field Notes: Workshop 2 (FNWS2) – Consolidation and Collective Direction**

**Analytic focus:** Group cohesion, legitimacy of student expertise, shared purpose

Workshop 2 opened with a structured recap of priorities identified through dot voting in Workshop 1. This explicit acknowledgement of prior student input appeared to function as a legitimising act. Students immediately recognised artefacts from the previous session and referenced them without prompting (e.g., “This was our point about PPE waste”), indicating continuity and ownership of the process.

Group dynamics differed noticeably from Workshop 1. Students spoke more readily, interruptions were collaborative rather than competitive, and disciplinary identity was invoked as a resource rather than a boundary (e.g., “From pharmacy we see this a lot…”). Mixed-discipline groupings led to visible moments of comparison and synthesis, particularly during brainstorming around “Where does the resource start and end?” Students physically moved between flipcharts, pointing across groups to align ideas, suggesting an emerging shared mental model of the resource.

Thematic clustering activities revealed how interdisciplinary dialogue shaped priorities. For example, content initially framed as “global” by one group was repositioned as “health impacts” following discussion, demonstrating negotiation rather than dominance of a single perspective. Field notes captured several moments where students explicitly deferred to peers (“That makes more sense actually”), indicating reduced defensiveness and increased trust.

Facilitator intervention was minimal during this workshop. Students self-assigned roles (e.g., note-taker, spokesperson) without instruction, and timekeeping emerged organically within groups. This contrasted with Workshop 1, where facilitators frequently prompted progression. The post-workshop focus group reflected this shift, with students describing the process as “flowing” and “more natural,” supporting the development of shared purpose and interprofessional alignment.

**Focused Ethnographic Field Notes: Workshop 3 (FNWS3) – From Ideas to Ownership**

**Analytic focus:** Confidence, leadership, translation into design decisions

Workshop 3 demonstrated the most pronounced shift in participant confidence and autonomy. The session opened with a recap and explicit reference to feedback from previous focus groups (e.g., name badges, clearer structure, preparatory materials). Students responded positively to these changes, with several commenting informally that it felt “more professional” or “easier to settle.”

During engagement with examples of gamified e-resources, students adopted an evaluative stance, critiquing design choices using pedagogical language (e.g., “too much clicking,” “that would overload people”). This represented a shift from earlier workshops, where students framed contributions cautiously. Here, critique was assertive and often collective, suggesting growing legitimacy of student expertise.

Design sprints generated sustained, animated discussion. Groups negotiated navigation logic, colour schemes, and interactive features with minimal facilitator input. Artefacts (storyboards, wireframes) were frequently revised in real time, with students crossing out, re-drawing, and annotating each other’s work. Field notes recorded moments where students explicitly linked design decisions back to earlier priorities (e.g., “We said it had to be practical, so this scenario works better”), evidencing coherent integration across workshops.

Dot voting in this workshop functioned differently than in Workshop 1. Rather than exploratory prioritisation, it was used to finalise preferences, with less debate and faster consensus. This suggested increased alignment and confidence in collective judgement. The post-workshop focus group reflected pride and anticipation, with students speaking about the resource as something they had “made” rather than “contributed to,” signalling strong ownership.
